# A GASTRIC ENIGMA: THE SECRET BEHIND THE UPPER GI BLEED

**DOI:** 10.51894/001c.144425

**Published:** 2025-09-30

**Authors:** Mohammad H. Ayoub

**Affiliations:** 1 College of Osteopathic Medicine Michigan State University

## 66

### INTRODUCTION

Trichobezoars are rare gastric masses formed from ingested hair, often associated with trichophagia and psychiatric conditions. They typically present with nonspecific gastrointestinal (GI) symptoms such as abdominal pain, nausea, vomiting, and early satiety. In rare cases, complications like gastric outlet obstruction, perforation, or upper GI bleeding can occur. We report a 14-year-old female with hematemesis as the primary manifestation of a gastric trichobezoar, an uncommon presentation leading to its incidental discovery.

### CASE DESCRIPTION

A 14-year-old female presented with multiple episodes of hematemesis over one day. She had stable vital signs but significant anemia, with hemoglobin dropping from 10.5 g/dL to 8.6 g/dL in under 24 hours. Examination revealed pale conjunctiva and a soft, non-tender abdomen. Laboratory results showed normal platelet levels and positive fecal occult blood. Her history included anxiety during the COVID-19 pandemic, briefly treated with Zoloft and counseling, and chronic headaches self-managed with daily Excedrin for one month.

The differential diagnosis included esophagitis, Mallory-Weiss tear, esophageal varices, gastric or duodenal ulcers, and gastritis. The patient was admitted, placed NPO, started on IV Protonix, and underwent esophagogastroduodenoscopy (EGD) to determine the bleeding source.

EGD revealed a Forrest III ulcer in the gastric antrum with low rebleeding risk. However, a large trichobezoar was discovered, occupying most of the gastric body and antrum. The duodenum was clear of hair, ruling out Rapunzel syndrome. The patient later admitted to trichophagia during a period of high anxiety two years prior. She underwent exploratory laparotomy for bezoar removal and was discharged on a proton pump inhibitor with psychiatric referral.

### DISCUSSION/CONCLUSIONS

Trichobezoars are rare and can present with nonspecific GI symptoms, often delaying diagnosis. In this case, hematemesis resulted from chronic mucosal pressure leading to ischemia and ulceration. While endoscopic removal is possible for small bezoars, surgery is required for larger ones. Psychiatric evaluation is essential to prevent recurrence. This case highlights the importance of considering trichobezoars in young patients with unexplained GI bleeding and emphasizes the need for a multidisciplinary approach.

